# E148Q variant: a familial Mediterranean fever-causing mutation or a sequence variant?

**DOI:** 10.1007/s00431-024-05690-5

**Published:** 2024-08-15

**Authors:** Elham Orouk Awaad, Lana Khoury, Joeri W. van Straalen, Adi Miller-Barmak, Tal Gazitt, Jumana Haddad-Haloun, Riva Brik, Mohamad Hamad Saied

**Affiliations:** 1 Department of Pediatrics, Lady Davis Carmel Medical Center, Haifa, Israel; 2 Department of Pediatric Immunology and Rheumatology, Wilhelmina Children’s Hospital, University Medical Center Utrecht, P.O. box 85090, 3508 AB Utrecht, the Netherlands; 3 Rheumatology Unit, Carmel Medical Center, Haifa, Israel; 4 Division of Rheumatology, Department of Medicine, University of Washington Medical Center, Seattle, WA USA; 5 Rappaport Faculty of Medicine, Technion-Israel Institute of Technology, Haifa, Israel; 6 Human Genetics Institute, Lady Davis Carmel Medical Center, Haifa, Israel

**Keywords:** Familial Mediterranean fever, FMF, p.E148Q, p.V726A, Druze, Ethnicity

## Abstract

Familial Mediterranean fever (FMF) is an autosomal recessive autoinflammatory disease, linked to mutations in the *MEFV* gene. The p.E148Q variant, found on exon 2, has an uncertain role in FMF, with debates on whether it is a benign polymorphism or a pathogenic mutation. This study aimed to assess the clinical characteristics and severity of FMF in patients homozygous for the p.E148Q variant and to evaluate the impact of the p.V726A variant in these patients. This retrospective cohort study analyzed data from electronic medical records at Carmel Medical Center, Israel. Patients who underwent genetic testing for FMF from November 2004 to December 2019 and had p.E148Q/p.E148Q or p.E148Q/p.E148Q + p.V726A variants were included. Disease severity was assessed using the Tel Hashomer Key to Severity Score. Statistical analyses compared clinical characteristics and severity between genotype groups. The study included 61 FMF patients, with 24 (39%) having p.E148Q/p.E148Q and 37 (61%) having p.E148Q/p.E148Q + p.V726A variants. The majority (72%) were Druze. Most patients (65.5%) exhibited mild disease, while 31.1% had moderate disease, with no cases of severe disease. Colchicine treatment significantly reduced CRP levels in all patients.

*Conclusion*: These findings suggest that the p.E148Q variant, whether alone or with p.V726A, generally results in mild to moderate FMF severity, supporting its pathogenic role in particular ethnicity. These results contribute to understanding the clinical significance of the p.E148Q variant and considering the patient’s need for Colchicine treatment.

**Table:** 

**What is Known:** *• The role of the p.E148Q variant in FMF is debated, with questions about whether it is a benign polymorphism or a pathogenic mutation.* *• The prevalence of MEFV variants can vary significantly among different ethnic groups.*	
**What is New:** *• The p.E148Q variant has clinical significance in particular ethnicities, as supported by a significant reduction in CRP levels following colchicine treatment.* *• The p.E148Q variant, whether alone or with p.V726A, generally results in mild to moderate FMF severity.*

**Supplementary information:**

The online version contains supplementary material available at 10.1007/s00431-024-05690-5.

## Introduction

Familial Mediterranean fever (FMF; OMIM 249100) is an inherited autosomal recessive autoinflammatory disease characterized by short, self-limited, and recurrent episodes of high fever accompanied by sterile serositis involving the abdomen, chest, and joints [1]. The disease is particularly common in populations of Turkish, Armenian, Jewish-Sephardic, and Arab descent [2].

The diagnosis of FMF is based on clinical symptoms and supported by genetic testing [3]. However, the absence of an appropriate mutation does not preclude the existence of the disease. Proper and early diagnosis is important because early treatment with colchicine can prevent symptomatic attacks and a late but major complication of the disease—systemic amyloidosis [4].

FMF is caused by mutations in the *MEFV* gene, found on human chromosome 16. This gene encodes the pyrin protein, which plays an important role in the regulation of the inflammatory response. The mutant pyrin results in the activation of the pyrin inflammasome, leading to an increase in inteleukine-1β (IL-1β) production [5, 6]. To date, 399 sequence variants are reported in the *MEFV* gene according to the INFEVERS database [7]. The most common pathogenic variants are on exon 10 (p.M680I, p.M694V, p.M694I, and p.V726A). Another common variant is p.E148Q, found on exon 2, where glutamine (Q) substitutes for glutamic acid (E), and is considered the most common homozygous variant in patients of Druze origin [2, 8].

There is considerable FMF genotype–phenotype variability; for instance, the p.M694V mutation is associated with a severe phenotype and the onset of renal AA amyloidosis, while the p.V726A mutation is associated with a milder form of the disease [9, 10].

One of the debated topics in FMF research is whether the p.E148Q variant is a benign polymorphism or a disease-causing mutation. This controversy has significant implications for the diagnosis and management of FMF, as the classification of p.E148Q can influence treatment decisions and genetic counselling. Therefore, the main objective of our study was to evaluate the clinical characteristics and disease severity associated with a homozygous p.E148Q variant in different ethnic groups of Arab origin, including Christians, Muslims, and Druze, in addition to Jewish patients. A secondary goal was to examine the contribution of a single allele of the p.V726A mutation to disease severity in homozygous p.E148Q patients, given the high prevalence of this mutation in our FMF patients, and to assess the impact of a well-known FMF mutation within our cohort.

## Methods

### Study design and source of data

This retrospective, long-term follow-up cohort study was conducted at Carmel Medical Center in Haifa, affiliated with Clalit Health Services (CHS), the largest healthcare provider in Israel. The study utilized data extracted from the electronic medical records of CHS/Carmel Medical Center.

CHS serves a diverse membership across Israel, encompassing a wide range of geographic locations, ethnicities, and socioeconomic backgrounds. CHS maintains comprehensive electronic medical records that are continuously updated with information from pharmaceutical, medical, and administrative sources.

### Ethical approval

The study was approved by the Institutional Review Board of Carmel Medical Center (CMC-0009–20) and conducted in accordance with the Declaration of Helsinki. The requirement for individual patient consent forms was waived by the Institutional Review Board due to the retrospective, observational nature of the de-identified data.

### Data collection

Data were extracted from CHS’s electronic medical records, including demographic, laboratory, and clinical information. This encompassed patient age, sex, ethnicity, medical history, disease onset, symptoms, frequency and duration of attacks, laboratory tests such as C-reactive protein (CRP) levels, and colchicine treatment and dosage.

### Inclusion criteria

All patients who underwent genetic testing for FMF at the hospital’s genetic institute between November 2004 and December 2019 were eligible for inclusion in the study.

### Exclusion criteria

Patients with variants other than p.E148Q/p.E148Q or p.E148Q/p.E148Q + p.V726A, as well as those with missing data or information, were excluded from the study.

### Disease severity assessment

Disease severity was assessed using the Tel Hashomer Key to Severity Score [3] for each patient (see Supplementary Table 1).

### Genetic analysis

For FMF genotyping, samples were tested for six variants using PRONTO FMF Screen kit (Pronto Diagnostics Ltd, Israel), which is a single-nucleotide primer extension enzyme-linked immunosorbent assay (ELISA) intended for the qualitative, in vitro detection of p.M680I (c.2040 G > C and c.2040 G > A), p.M694V (c.2080 A > G), p.M694I (c.2082 G > A), p.V726A (c.2177 T > C), and p.E148Q (c.442G > C) variants in the *MEFV* gene from November 2004 until December 2015. NanoChip technology (Savyon Diagnostics Ltd, Israel) was used for FMF genetic testing from January 2016 until the termination of the study. NanoChip technology encompasses an automated platform capable of detecting multiple targets for individual samples and analyzing multiple samples on the same electronic microarray. This method was used for detecting 12 *MEFV* (NM_000243.2) variants: c.442G > C (p.E148Q) in exon 2, c.1105C > T (p.P369S) in exon 3, and c.1437C > G (p.F479L) in exon 5; as well as the following variants on exon 10: c.2040G > C (p.M680I), c.2040G > A (p.M680I), c.2076_2078del (p.I692del), c.2080A < G (p.M694V), c.2082G > A (p.M694I), c.2084A > G (p.K695R), c.2177T > C (p.V726A), c.2230G > T (p.A744S), and c.2282G > A (p.R761H).

### Outcome measures

The primary outcome measure was FMF disease severity as measured by the Tel Hashomer Key to Severity Score among patients homozygous for the p.E148Q variant. The secondary outcome was to compare the severity score in patients homozygous for the p.E148Q variant with or without additional p.V726A mutation.

### Statistical analysis

Characteristics were compared between FMF patients with p.E148Q/p.E148Q and p.E148Q/p.E148Q + p.V726A/- *MEFV* variants. The Mann–Whitney *U* test was used to compare numerical variables, and the chi-square test and Fisher’s exact tests were used to compare categorical variables, as appropriate. Characteristics were furthermore compared between patients with mild and moderate disease severity according to the Tel Hashomer Key to Severity Score utilizing the Mann–Whitney *U* test and the chi-square and Fisher’s exact tests, as noted above. CRP levels before and after treatment were compared using the Wilcoxon signed-rank test.

All data were analyzed using R version 4.0.3 (R Foundation for Statistical Computing, Vienna, Austria). In all analyses, *p* ≤ 0.05 for the two-tailed tests was considered statistically significant.

## Results

A total of 134 patients with the p.E148Q homozygous variants were initially enrolled in the study, of which 70 patients with combined homozygous variants (homozygous variants for p.E148Q along with another known homozygous variant for FMF) were excluded. Three patients had missing information and were also excluded. A total of 61 FMF patients were included in the current study of which 24 (39%) had p.E148Q/p.E148Q variant and 37 (61%) had p.E148Q/p.E148Q + p.V726A/- variants. Of all included patients, the median age at FMF onset was 16.0 years (IQR, 8.5–29.5), and 26 (43%) of the patients were male. The largest ethnic group was Druze (*n* = 44, 72%), followed by Muslims (*n* = 12, 20%). Regarding the p.E148Q/p.E148Q + p.V726A/- *MEFV* variant, it is most prevalent in Druze 34 (91.9%). The baseline characteristics of the two genotype groups are summarized in Table 1.

**Table 1:** Characteristics of total cohort (*n* = 61) with p.E148Q and p.V726A variants

Variable	p.E148Q/p.E148Q (*n* = 24)	p.E148Q/p.E148Q + p.V726A/- (*n* = 37)	*P*-value
Male sex	11 (45.8%)	15 (40.5%)	0.89
Descent, *n* (%)			< 0.01*
*Christian*	1 (4.2%)	0 (0.0%)	
*Druze*	10 (41.7%)	34 (91.9%)	
*Jewish*	4 (16.7%)	0 (0.0%)	
*Muslim*	9 (37.5%)	3 (8.1%)	
Disease-onset (years), median (IQR), no. patients (*n*)	13.0 (7.0–24.5) *n* = 23	17.5 (9.0–31.25) *n* = 32	0.21
Colchicine dosage (mg/day), median (IQR), no. patients (*n*)	1.0 (1.0–2.0) *n* = 22	1.0 (1.0–1.5) *n* = 31	0.58
Attacks per month, median (IQR), no. patients (*n*)	2.0 (1.0–2.5) *n* = 15	1.0 (0.5–2.0) *n* = 21	0.06
CRP pre-treatment, (mg/dL), median (IQR), no. patients (*n*)	0.76 (0.11–3.00) *n* = 19	2.45 (0.91–16.00) *n* = 14	0.12
CRP post-treatment, (mg/dL), median (IQR), no. patients (*n*)	0.20 (0.11–0.80) *n* = 15	0.55 (0.14–1.90) *n* = 18	0.15
Tel Hashomer score, median (IQR)	5.0 (3.0–6.3)	4.0 (3.0–6.0)	0.21
**Severity, *n* (%)**			0.71
*Mild*	16 (66.7%)	24 (64.9%)	
*moderate*	8 (33.3%)	11 (29.7%)	
*No disease*	0 (0.0%)	2 (5.4%)	

*Statistically significant

*IQR* inter-quartile range, *n* number of patients

The severity of FMF, assessed by using the Tel Hashomer criteria [3] which was fulfilled by all patients but 2, was generally mild to moderate in both genotype groups. Forty (65.5%) patients had mild disease while 19 (31.1%) of the patients had moderate disease. Two (3.2%) patients had no disease. There were no patients with this variant with severe disease. There was no significant difference in the distribution of severity scores between the two genotype groups (*p* = 0.71, Table 1).

When stratified by disease severity (Table 2), patients with moderate disease had an earlier age at disease onset (median 9.0 years vs. 18.0 years, *p* = 0.03) and required higher colchicine doses (median 1.8 mg/day vs. 1.0 mg/day, *p* < 0.01) compared to those with mild disease. Additionally, patients with moderate severity experienced more frequent attacks (median 2.0 per month vs. 1.0 per month, *p* = 0.04) as expected. Disease severity did not differ significantly between the two *MEFV* variant groups.

**Table 2:** Characteristics of FMF patients (*n* = 59) according to disease severity

Variable	Mild severity (*n* = 40)	Moderate severity (*n* = 19)	*P*-value
Male sex	19 (47.5%)	6 (31.6%)	0.38
Descent, *n* (%)			0.71
*Christian*	1 (2.5%)	0 (0.0%)	
*Druze*	30 (75.0%)	12 (63.2%)	
*Jewish*	2 (5.0%)	2 (10.5%)	
*Muslim*	7 (17.5%)	5 (26.3%)	
Disease-onset (years), median (IQR), no. patients (*n*)	18.0 (10.5–31.0) *n* = 35	9.0 (3.5–24.0) *n* = 19	0.03*
Colchicine dosage (mg/day), median (IQR), no. patients (*n*)	1.0 (1.0–1.5) *n* = 35	1.8 (1.5–2.4) *n* = 18	< 0.01*
Attacks per month, median (IQR), no. patients (*n*)	1.0 (0.5–2.0) *n* = 19	2.0 (1.0–3.0) *n* = 15	0.04*
CRP pre-treatment, (mg/dL), median (IQR), no. patients (*n*)	1.1 (0.2–7.5) *n* = 20	2.3 (0.5–5.0) *n* = 13	0.63
CRP post-treatment, (mg/dL), median (IQR), no. patients (*n*)	0.3 (0.1–1.0) *n* = 18	0.2 (0.1–1.5) *n* = 15	0.88
MEFV mutation, *n* (%)			1.00
*E148Q/E148Q*	16 (40.0%)	8 (42.1%)	
*E148Q/E148Q* + *V726A/-*	24 (60.0%)	11 (57.9%)	

*Statistically significant

*IQR* inter-quartile range, *n* number of patients

**Fig. 1:**
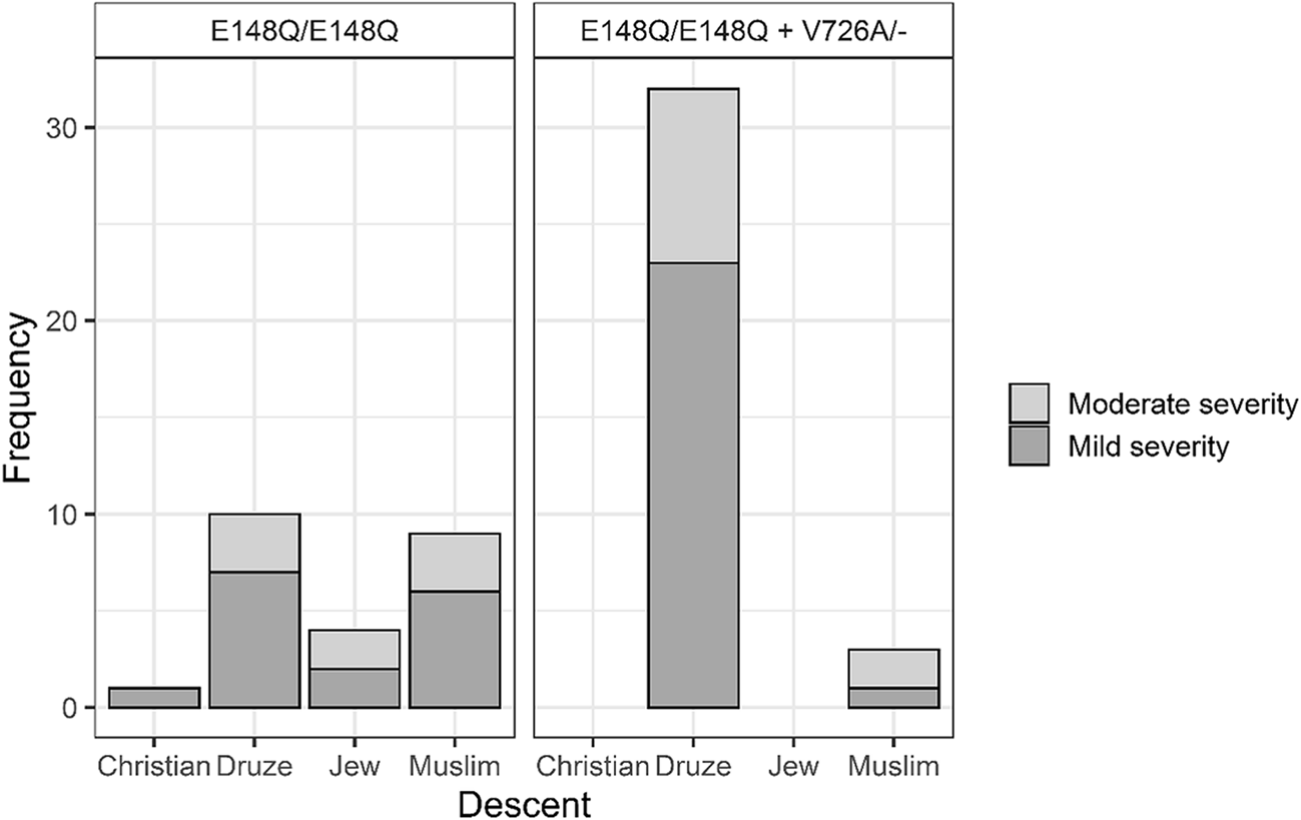
Stacked bar plots of disease severity in FMF patients (*n* = 59) of different descent, stratified by MEFV mutation

FMF patients of all ethnic groups had mild disease severity more frequently than moderate severity, except for Muslim patients with the p.E148Q/p.E148Q + p.V726A/- *MEFV* variant, whose disease severity was most commonly moderate (Fig. 1).

**Fig. 2:**
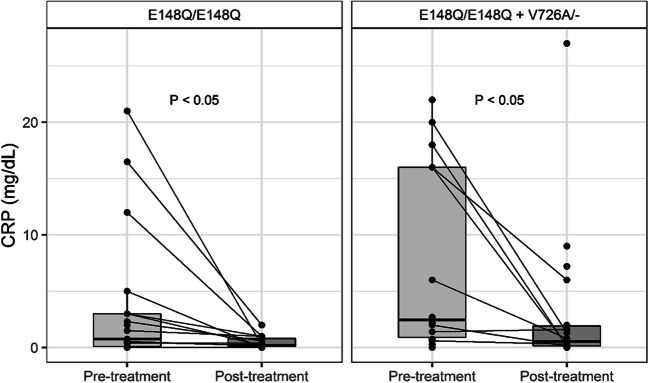
Paired boxplots of CRP levels before and after colchicine treatment, stratified by MEFV mutation

In addition, we noted that colchicine treatment has led to a significant reduction in CRP levels in all homozygous patients to p.E148Q/p.E148Q variant whether they had additional p.V726A mutation or not (Fig. 2 depicts the changes in CRP levels before and after colchicine treatment, stratified by *MEFV* genotype). However, in the presence of p.V726A mutation, the colchicine dosage was higher (Table 2).

## Discussion

Over the years, FMF research has been fraught with debate on whether the pure p.E148Q variant is an insignificant variant [11–14], a disease-causing variant with low penetrance and mild symptoms [15], or a significant variant in specific ethnic groups [16, 17]. This study aimed to clarify the clinical characteristics and disease severity associated with the p.E148Q variant in FMF patients and contribute to the ongoing debate regarding its pathogenicity.

Our results demonstrated that the p.E148Q variant, whether in the homozygous state (p.E148Q/p.E148Q) or combined with the p.V726A mutation (p.E148Q/p.E148Q + p.V726A), is generally associated with mild to moderate disease severity according to Tel Hashomer Key to Severity Score [3]. Specifically, 40/59 = 67.7% of the patients exhibited mild disease, while 19/59 = 32.2% had moderate disease, and no patients presented with severe disease. This supports the hypothesis that patients who are homozygous for the p.E148Q variant with or without p.V726A may display a clinical FMF phenotype with mild to moderate severity.

Previous studies have yielded mixed results regarding the significance of the p.E148Q variant. For instance, a genetic study from Israel on patients of Jewish-Sephardic descent found similar frequencies of the p.E148Q allele among patients and controls, suggesting it might be a benign polymorphism [11]. Another study from Israel could not provide evidence to support the notion that p.E148Q is a disease-causing variant and not a mere genetic polymorphism, despite the large sample size [18]. Conversely, Aydin et al. reported that Turkish FMF patients with the p.E148Q variant alone presented later in life and had a milder disease course but exhibited similar clinical findings to those with more severe variants [15]. Similarly, Topaloglu et al. found that Turkish patients who are homozygous for p.E148Q and negative for other pathogenic *MEFV* variants may display a clinical FMF phenotype with moderate to severe disease activity, although the severity may be milder compared to patients with other variants [17].

A study on Egyptian FMF patients uniquely found the p.E148Q variant to be the most frequent *MEFV* variant (38.6%) in their FMF cohort, associating this variant with abdominal pain, fever, and high serum amyloid A. Notably, in this study, all patients were of Arabic descent, and 57.2% of this group had undefined variants [16].

Our current study strengthens these findings from several cohorts worldwide, suggesting that the p.E148Q variant is associated with a clinical phenotype rather than being a benign variant. The differences observed in different cohorts likely result from the varying prevalence of variants among different ethnic groups. In Israel, while the homozygous p.M694V mutation is the most prevalent among Jews, the p.E148Q variant is the most common among Druze (56%). Among other Arab citizens in Israel (Muslims and Christians), the three most prevalent homozygous mutations are p.V726A, p.M694V, and p.M694I [8]. This may explain the high percentage of Druze patients homozygous for the p.E148Q variant in our current study (72%) in addition to their significant geographical distribution in our region.

In a recent article, Ben-Chetrit et al. discuss the significance of carrying MEFV variants in symptomatic and asymptomatic individuals, since many MEFV gene variants remain unclassified, with around 30% as variants of uncertain significance (VUS). They conclude that only the presence of likely pathogenic variants on both alleles should result in a definitive diagnosis. Otherwise, diagnoses such as probable FMF or FMF-like disease are considered. Additionally, they emphasize that genetic testing is only part of the diagnostic process; a comprehensive evaluation of a patient’s medical history and clinical symptoms is essential for accurate diagnosis and treatment [19].

Adding to the ethnic diversity, the overall disease severity in our study was mild to moderate across all ethnic groups, except for Muslim patients with the p.E148Q/p.E148Q + p.V726A variant, who exhibited predominantly moderate disease severity. This ethnic variability underscores the importance of considering genetic background, in addition to the environmental factors, when evaluating FMF patients.

It is generally known that the addition of another heterozygous variant typically causes more serious disease phenotype in FMF. For instance, the p.V726A ± p.E148Q allele is considered to be associated with severe disease and strongly predisposes to renal amyloidosis, in contrast to an isolated p.E148Q variant [20]. Also, patients homozygous for the complex allele p.E148Q-p.V726A/ p.E148Q-p.V726A, or p.E148Q-p.V726A/ p.V726A compound heterozygotes have more severe disease compared to patients homozygous for p.V726A [18]. Interestingly, in our study, the addition of the p.V726A mutation did not aggravate the clinical phenotype in our cohort. However, knowing that p.V726A is a pathogenic variant, it is possible that with an increased number of patients in the cohort, the number of patients with moderate disease and carrying the p.E148Q/p.E148Q + p.V726A genotype will be significantly higher than those affected with the mild form and homozygous for p.E148Q. The fact that p.E148Q variant may have clinical significance even without additional *MEFV* mutations is further supported by the significant reduction in CRP levels following colchicine treatment, confirming the pathogenicity of the p.E148Q variant through objective inflammatory markers. In addition, this finding supports the importance of identifying and treating also cases with a mild disease severity.

Our study also examined clinical parameters such as age at disease onset, frequency of attacks, and colchicine dosage. Patients with moderate disease had an earlier onset, more frequent attacks, and required higher doses of colchicine compared to those with mild disease. These findings align with the expected progression of FMF, where earlier onset and higher attack frequency indicate more active disease.

However, our study has several limitations. Its retrospective design and reliance on electronic medical records may not capture all relevant clinical details. Additionally, the genetic analysis did not include the full length of the *MEFV* gene, potentially missing other pathogenic variants. Future research should include larger, multicenter cohorts and comprehensive genetic testing to validate these results and explore the molecular mechanisms underlying the variable expressivity of the p.E148Q variant.

In conclusion, our study provides valuable insights into the clinical characteristics and disease severity associated with the p.E148Q variant in FMF patients, supporting its pathogenic role in particular ethnicity. The findings suggest that p.E148Q, whether alone or in combination with p.V726A, generally results in mild to moderate disease, responds well to colchicine treatment, and exhibits variability across different ethnic groups. Continued research is essential to fully elucidate the pathogenic nature of the p.E148Q variant and its implications for FMF diagnosis and treatment.

## Supplementary information

Below is the link to the electronic supplementary material.

Supplementary file1 (DOCX 24.5 KB)

## Data Availability

All relevant data are reported in the article. The data used and/or analyzed during the present study are available from the corresponding author on reasonable request.

## Abbreviations

*FMF*: Familial Mediterranean fever
*CHS*: Clalit Health Services
*HMOs*: Health Maintenance Organizations
*CRP*: C-reactive protein
