# Supplementary material for: E148Q variant: a familial Mediterranean fever-causing mutation or a sequence variant?
Source: Eur J Pediatr. 2024 Aug 15;183(10):4499–506. doi: 10.1007/s00431-024-05690-5 (PMC11413036; doi:10.1007/s00431-024-05690-5)
Supplement: Supplementary file 1 — Supplementary file1 (DOCX 24.5 KB) [file 431_2024_5690_MOESM1_ESM.docx]

**Supplementary Table 1: Tel Hashomer Key to Severity Score for FMF**

|  | **Points** |
| --- | --- |
| **Age of onset** |  |
| <5 years | 3 |
| 5-10 years | 2 |
| 10-20 years | 1 |
| >20 years | 0 |
| **Frequency of attacks** |  |
| >2 per month | 3 |
| 1-2 per month | 2 |
| <1 per month | 1 |
| **Colchicine dosage to control attacks** |  |
| Non-responders | 4 |
| 2 mg/day | 3 |
| 1.5 mg/day | 2 |
| 1 mg/day | 1 |
| **Arthritis** |  |
| Protracted arthritis | 3 |
| Presence of acute joints | 2 |
| **Erysipelas-like erythema** | 2 |
| **Amyloidosis** | 3 |
| **Phenotype II** | 4 |
|  | |

**NOTE.** Total of points indicates the following: mild disease, 2-5 points; moderate disease, 6-10 points; severe disease, >10 points(3).
